# Supplementary figures and images for: Neisseria gonorrhoeae co-opts C4b-binding protein to enhance complement-independent survival from neutrophils
Source: PLoS Pathog. 2023 Mar 2;19(3):e1011055. doi: 10.1371/journal.ppat.1011055 (PMC10013916; doi:10.1371/journal.ppat.1011055)

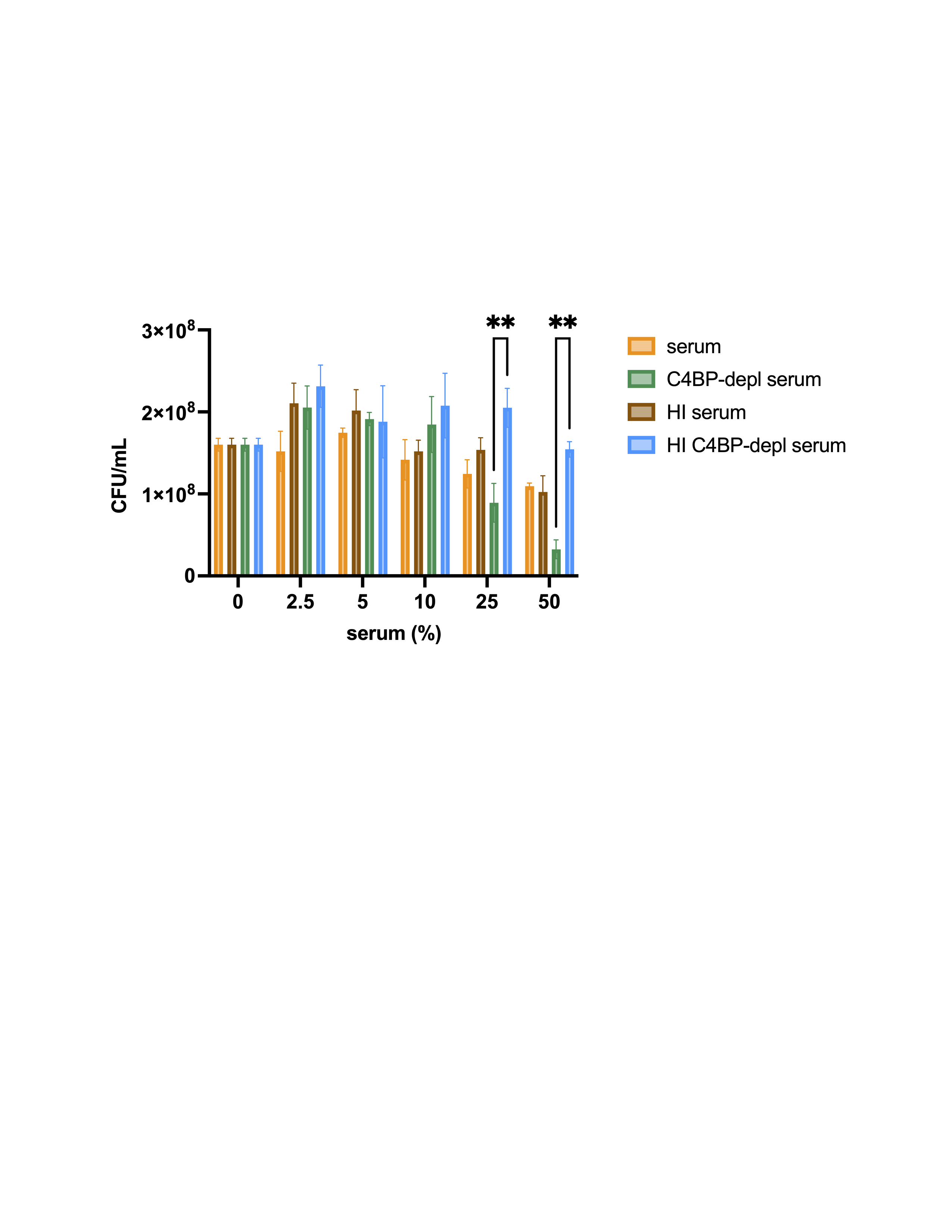

Supplement: S1 Fig — OpaD+ Gc (10^8 CFU/ml) was resuspended in the indicated percent of normal human serum (Lund University) or C4BP-depleted (depl) serum, with or without heat-inactivation (HI), for 20 minutes at 37°C. CFU were then enumerated from washed bacterial suspensions. Two-way ANOVA with Sidak’s post-hoc comparisons was used to compare across conditions at matched serum concentrations. Data represent the mean ± SEM of 3 independent experiments. **p<0.01. (TIF) [file ppat.1011055.s001.tif]

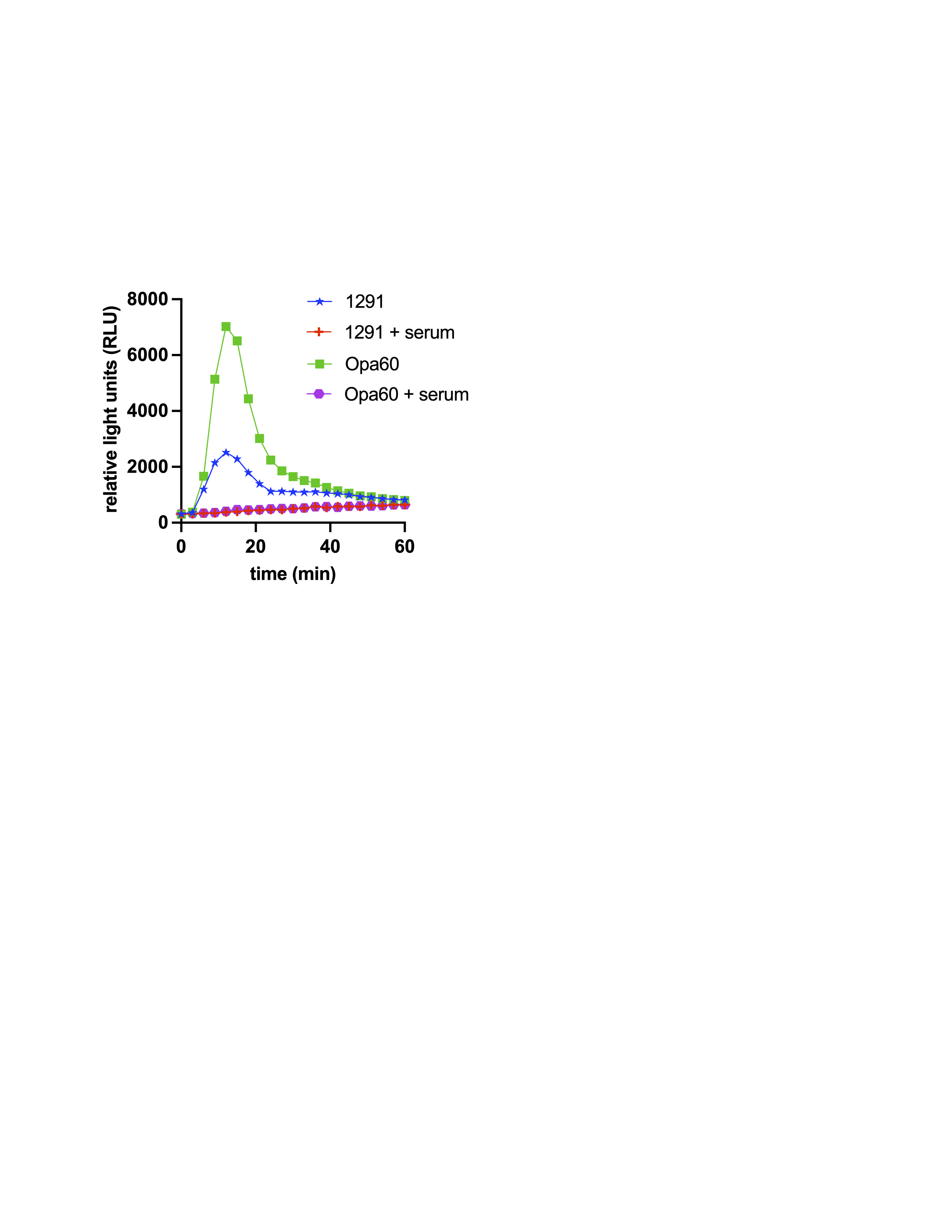

Supplement: S2 Fig — Strain FA1090 Gc constitutively expressing Opa60 (Opa60+) and an undefined Opa+ isolate of strain 1291 Gc were incubated with normal human serum (UVA) or left untreated, then exposed to primary human neutrophils. ROS production was measured as in Fig 1C. Results are one representative of 3 independent experiments. (TIF) [file ppat.1011055.s002.tif]

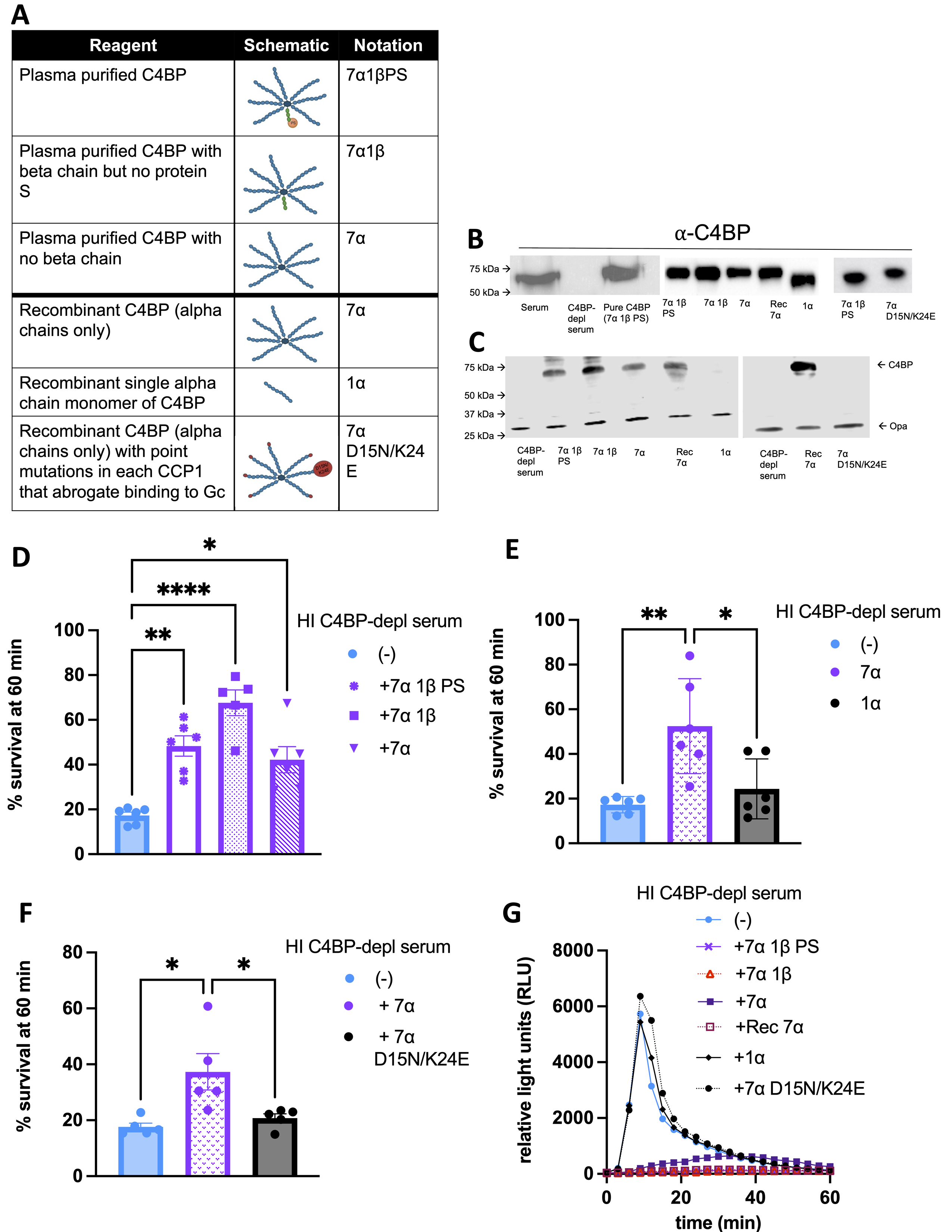

Supplement: S4 Fig — (A) Schematic and description for each C4BP species used in (B-G). (B) The indicated form of C4BP or sera (Lund University) were separated by SDS-PAGE and subjected to Western blot for C4BP (rabbit anti-C4BPA IgG, Novus). (C) OpaD+ Gc was incubated in heat-inactivated C4BP-depleted serum, alone or with each indicated form of C4BP added back. Bacterial lysates were resolved on a 4–20% gradient gel C4BP in each lysate was detected as in (B). Anti-Opa 4B12 mAb served as a loading control. Blots were developed by LI-COR Odyssey. (D-F) OpaD+ Gc was incubated in 25% heat-inactivated C4BP-depleted serum alone (“(-)”) or to which the indicated species as defined in (A) were added back (50 μg/mL). Bacterial survival after 60 minutes of exposure to neutrophils was measured as in Fig 1A, for 3–4 independent experiments. One-way ANOVA followed by Tukey’s post-hoc comparisons was used to compare each condition to C4BP-depleted serum alone. (G) OpaD+ Gc was incubated with the indicated species of C4BP added to heat-inactivated C4BP-depleted serum as in (F). Neutrophil ROS production in response to each condition was measured as in Fig 1C. Results are one representative of 3 independent experiments. *p<0.05, **p<0.01,****p<0.0001. (TIF) [file ppat.1011055.s004.tif]

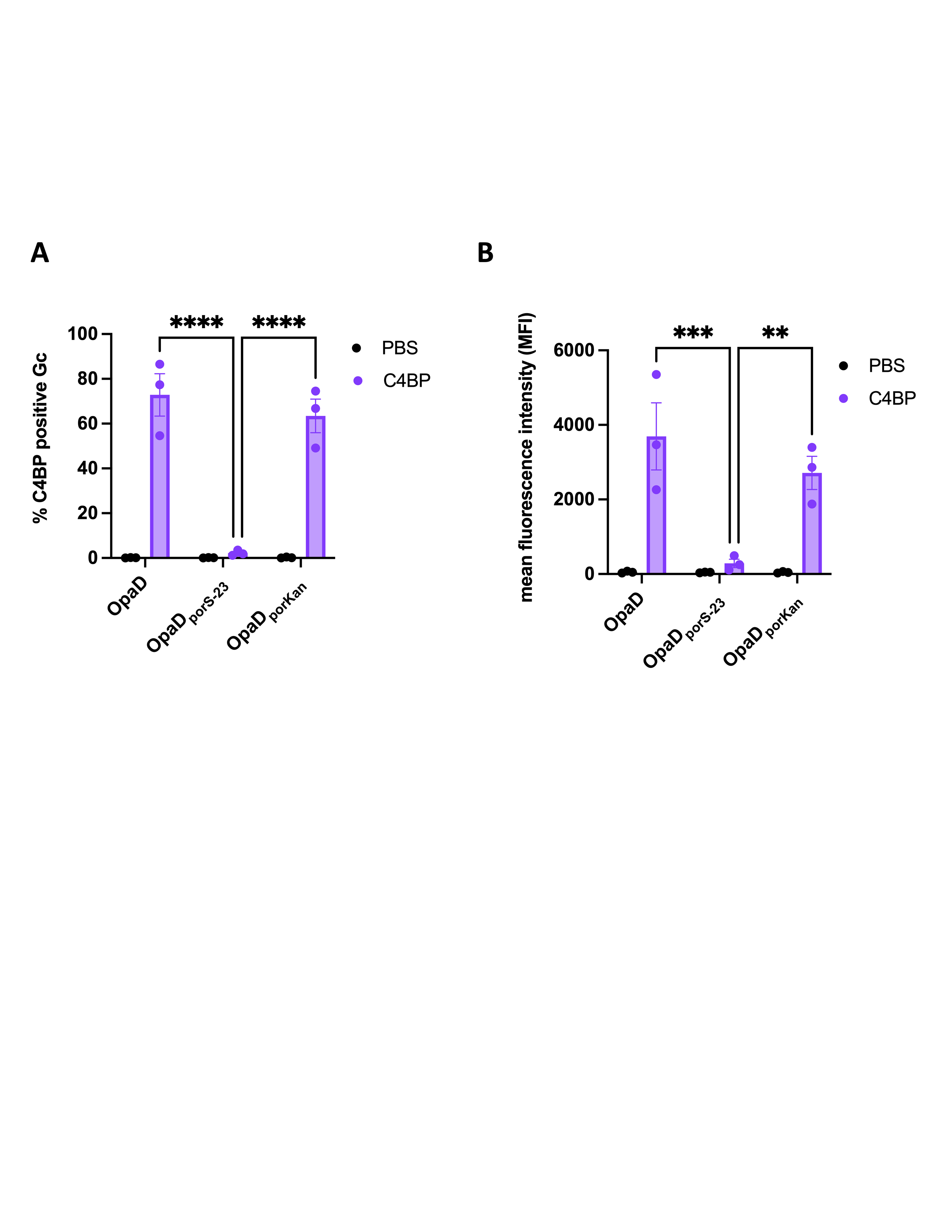

Supplement: S5 Fig — OpaD+ Gc, OpaDporS-23 Gc, and OpaDporKan Gc were incubated with C4BP (50 μg/ml) or left untreated (PBS). C4BP bound to the surface of the bacteria was measured by imaging flow cytometry as in Fig 3B and 3C. (B), percent C4BP-positive bacteria; (C), mean intensity of AF488 fluorescence of the total bacterial population. Results are the mean ± SEM from 3 independent experiments. Statistics are performed using two-way ANOVA with Sidak’s post-hoc comparisons to OpaDporS-23 Gc incubated with C4BP. **p<0.01,***p<0.001, ****p<0.0001. (TIF) [file ppat.1011055.s005.tif]

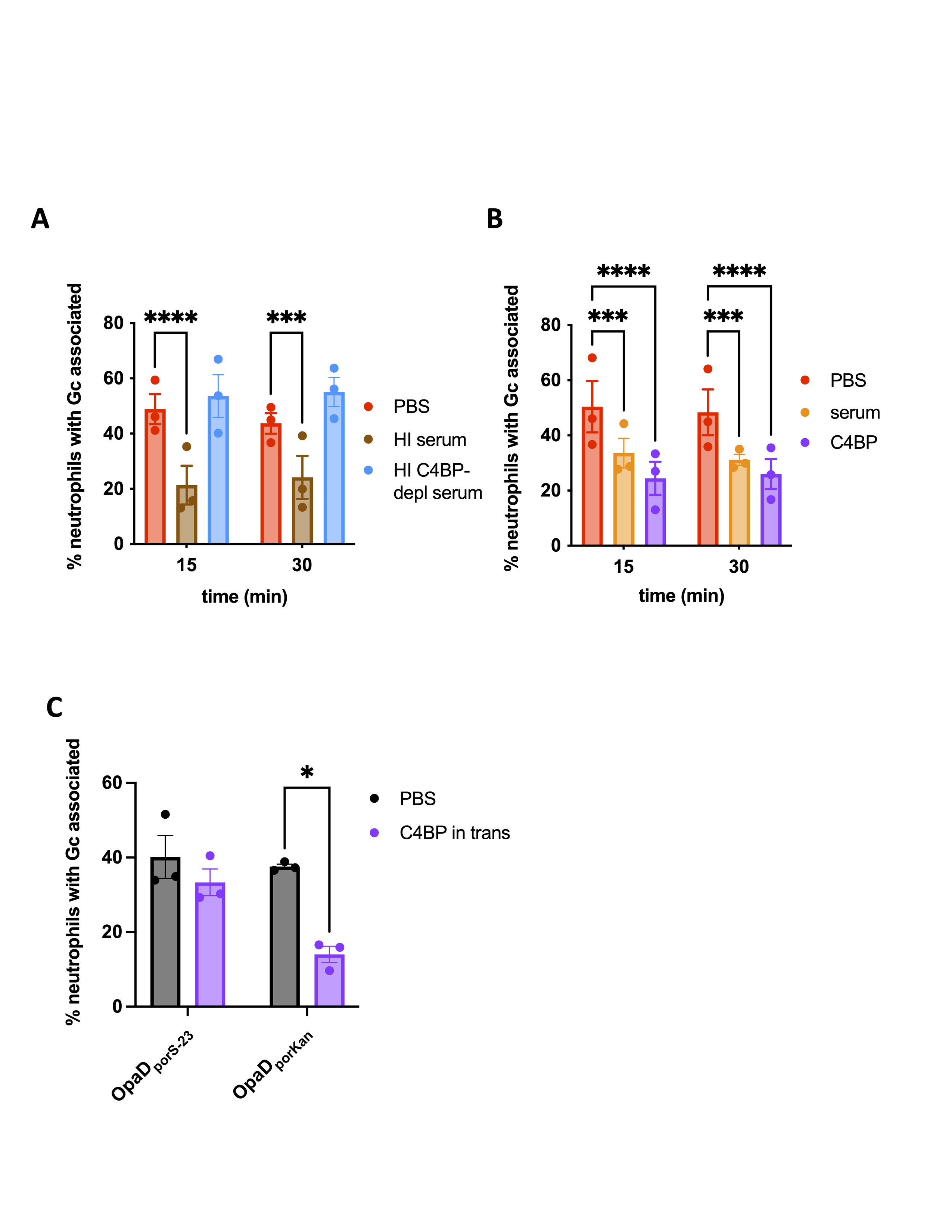

Supplement: S6 Fig — (A-B) OpaD+ Gc was incubated in PBS (red), C4BP-replete serum (Lund University) that was not heated (orange) or was heat inactivated (brown), C4BP-depleted heat-inactivated serum (blue), or purified C4BP (purple). Gc was then exposed to neutrophils, and the percentage of neutrophils with ≥1 associated bacterium was measured as in Fig 5B. Statistics were performed by two-way ANOVA followed by Sidak’s multiple comparisons for data from 3 independent experiments. Results in (A) and (B) are from the same infected cell population as shown in Fig 5D and 5E, respectively, for phagocytosed Gc. (C) OpaDporS-23 Gc and OpaDporKan Gc were incubated with neutrophils, with (purple) or without (grey) C4BP added to the medium immediately prior to infection (“in trans”). The percentage of neutrophils with ≥1 associated bacterium was measured as in Fig 5B. Statistics were performed by two-way ANOVA followed by Sidak’s multiple comparisons for data from 3 independent experiments. *p<0.05, ***p<0.001, ****p<0.0001. Results in (C) are from the same infected cell population as in Fig 5G for phagocytosed Gc. (TIF) [file ppat.1011055.s006.tif]

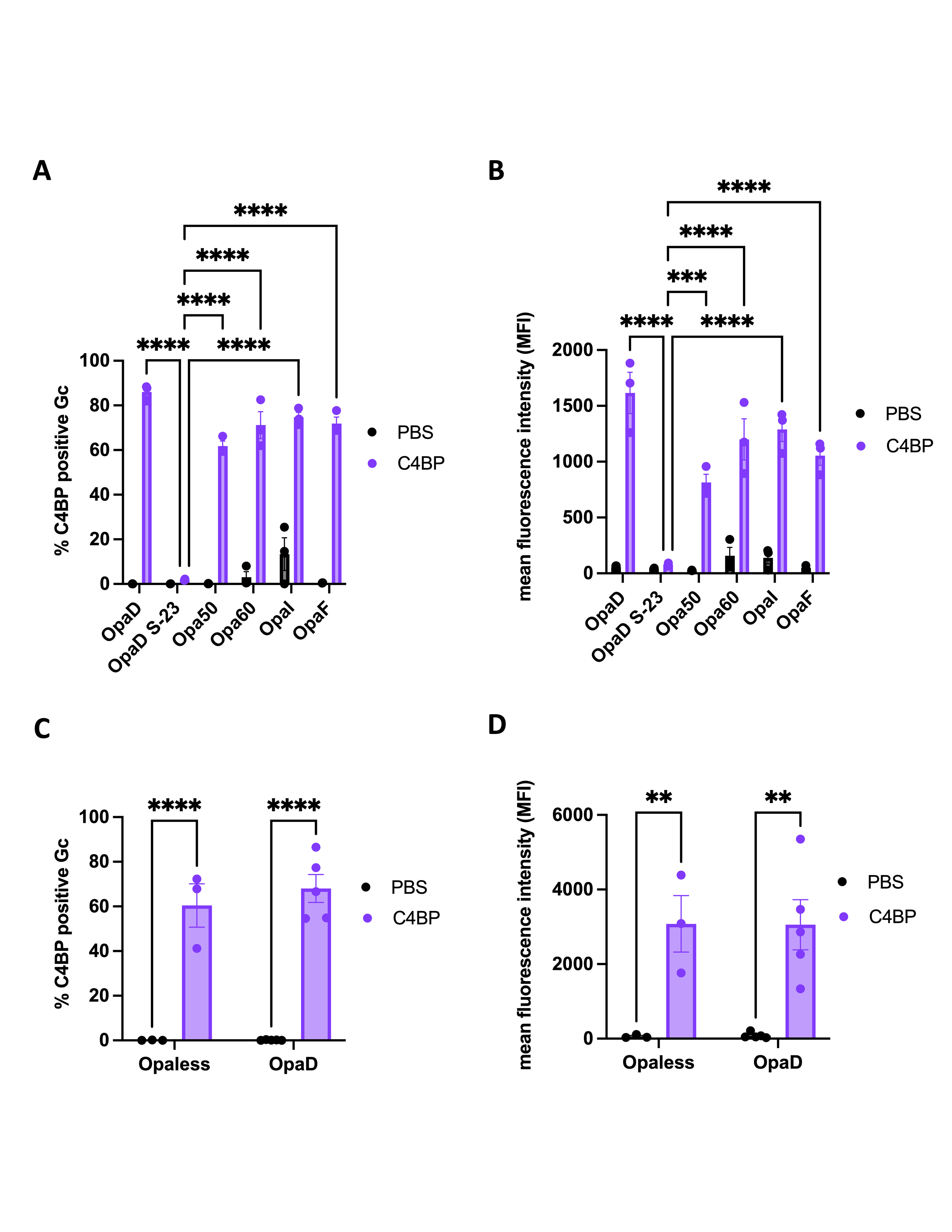

Supplement: S7 Fig — The indicated variants or mutants of Gc were incubated in C4BP or PBS, then processed for imaging flow cytometry as in Fig 3. OpaD+ Gc and OpaDporS-23 Gc are positive and negative controls for C4BP binding, respectively. (A,C) Percentage of C4BP-positive bacteria; (B,D) mean intensity of C4BP (AF488) fluorescence of the total bacterial population. Statistical analyses in (A) and (B) are performed by two-way ANOVA with Sidak’s post-hoc comparisons to OpaDporS-23 Gc incubated with C4BP. Statistics in (C) and (D) are performed by two-way ANOVA with Sidak’s post-hoc comparisons to untreated Gc. Results are the mean ± SEM of 3 independent experiments. **p<0.01,***p<0.001, ****p<0.0001. (TIF) [file ppat.1011055.s007.tif]

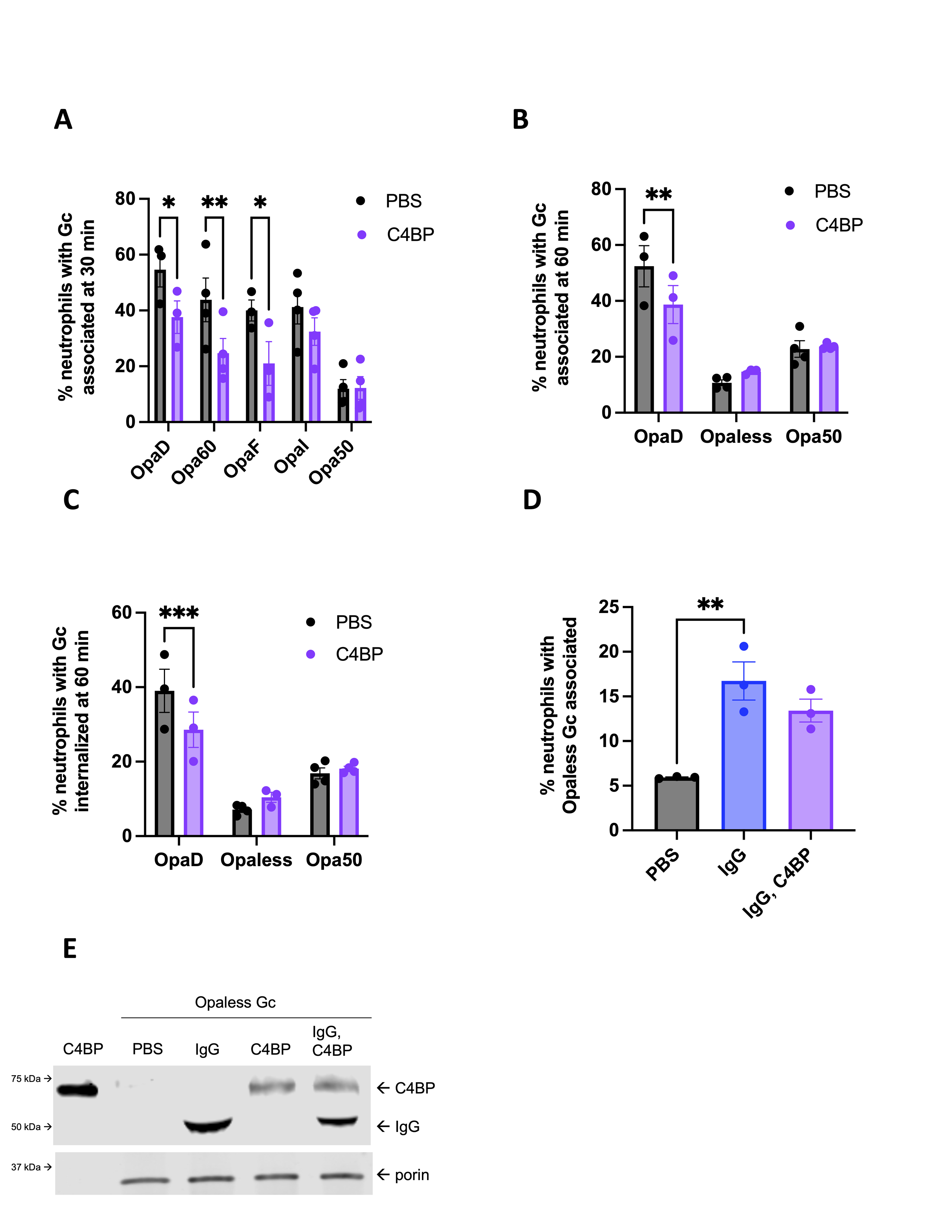

Supplement: S8 Fig — (A-C) The indicated variants of Gc were incubated in C4BP (50 μg/ml, purple) or PBS (grey) as in Fig 6A. The bacteria were incubated with neutrophils for 30 minutes (A) or 1 hour (B,C), then processed for imaging flow cytometry as in Fig 5. (A,C) report the percentage of neutrophils with ≥1 associated Gc as in Fig 5B, and (B) reports the percentage of neutrophils with ≥ 1 phagocytosed Gc as in Fig 5C. Results are the mean ± SEM from ≥3 independent experiments. Statistical analyses were performed by two-way ANOVA followed by Sidak’s multiple comparisons. Data in (A) are from the same infected cell population as in Fig 6A for phagocytosed Gc. (D-E) Opaless Gc was incubated with C4BP, opsonized in rabbit anti-Gc IgG, sequentially incubated with IgG then C4BP, or left untreated as in Fig 6B. (D) The bacteria were incubated with neutrophils for 30 minutes, then processed for imaging flow cytometry as in A. Statistics were performed by one-way ANOVA followed by Tukey’s multiple comparisons. Data in (D) are from the same infected cell population as in Fig 6B for phagocytosed Gc. (E) C4BP in bacterial lysates was detected as in S4 Fig, except anti-porin H5.2 antibody served as the loading control. *p<0.05, **p<0.01,***p<0.001. (TIF) [file ppat.1011055.s008.tif]

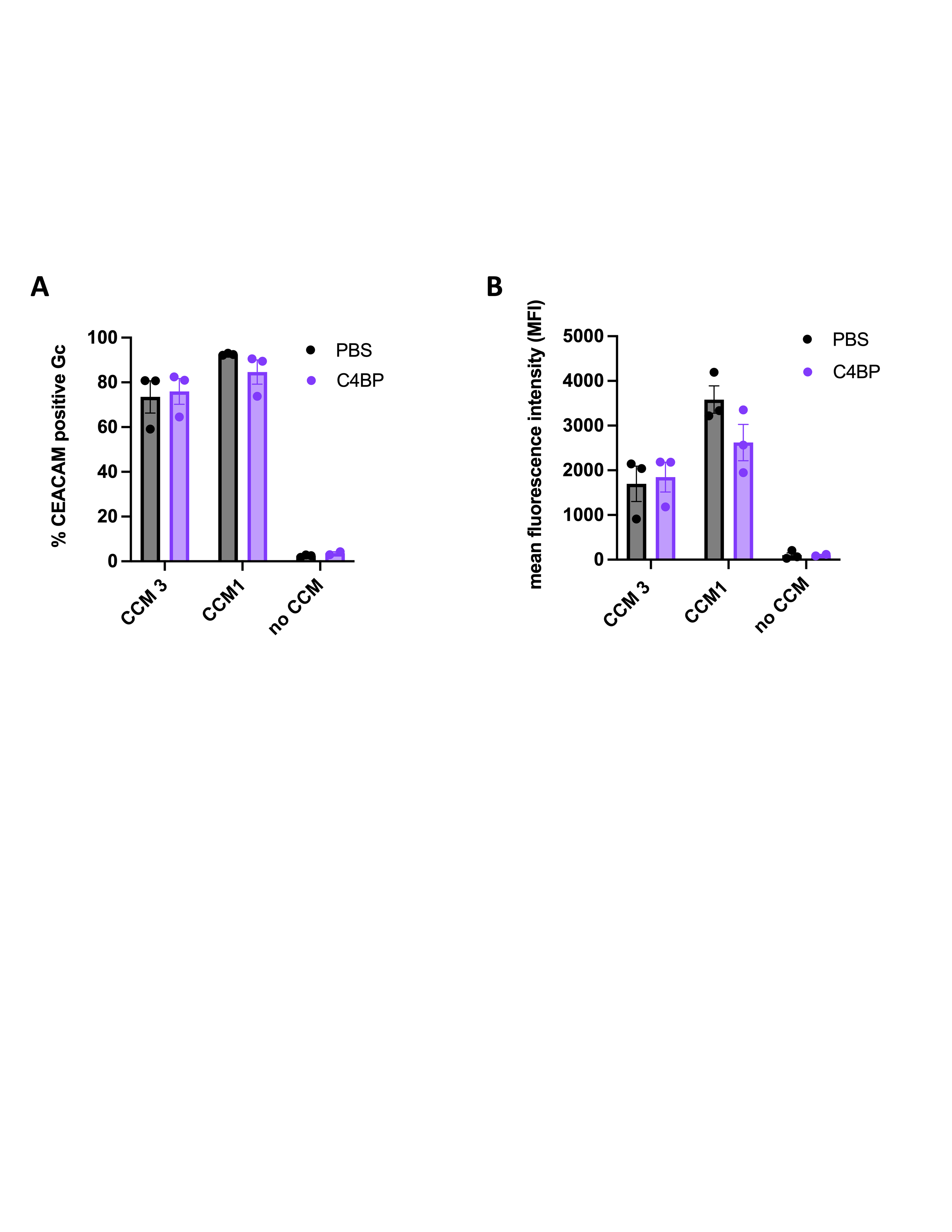

Supplement: S9 Fig — (A-B) OpaD+ Gc was incubated with C4BP (purple) or in PBS (gray), washed, and mixed with GST-tagged recombinant N-terminal domains of CEACAM-1 (CCM 1) or CEACAM-3 (CCM 3), or in buffer (no CCM). N-CEACAM precipitation with Gc was detected with an anti-GST antibody followed by Alexa Fluor 488-coupled goat anti-mouse IgG, and analyzed by imaging flow cytometry. (A) Percent CEACAM-positive bacteria; (B) mean intensity of CEACAM (AF488) fluorescence of the total in focus, singlet bacterial population. Results are the mean ± SEM from 3 independent experiments. Statistics were performed by two-way ANOVA followed by Sidak’s multiple comparisons. (TIF) [file ppat.1011055.s009.tif]

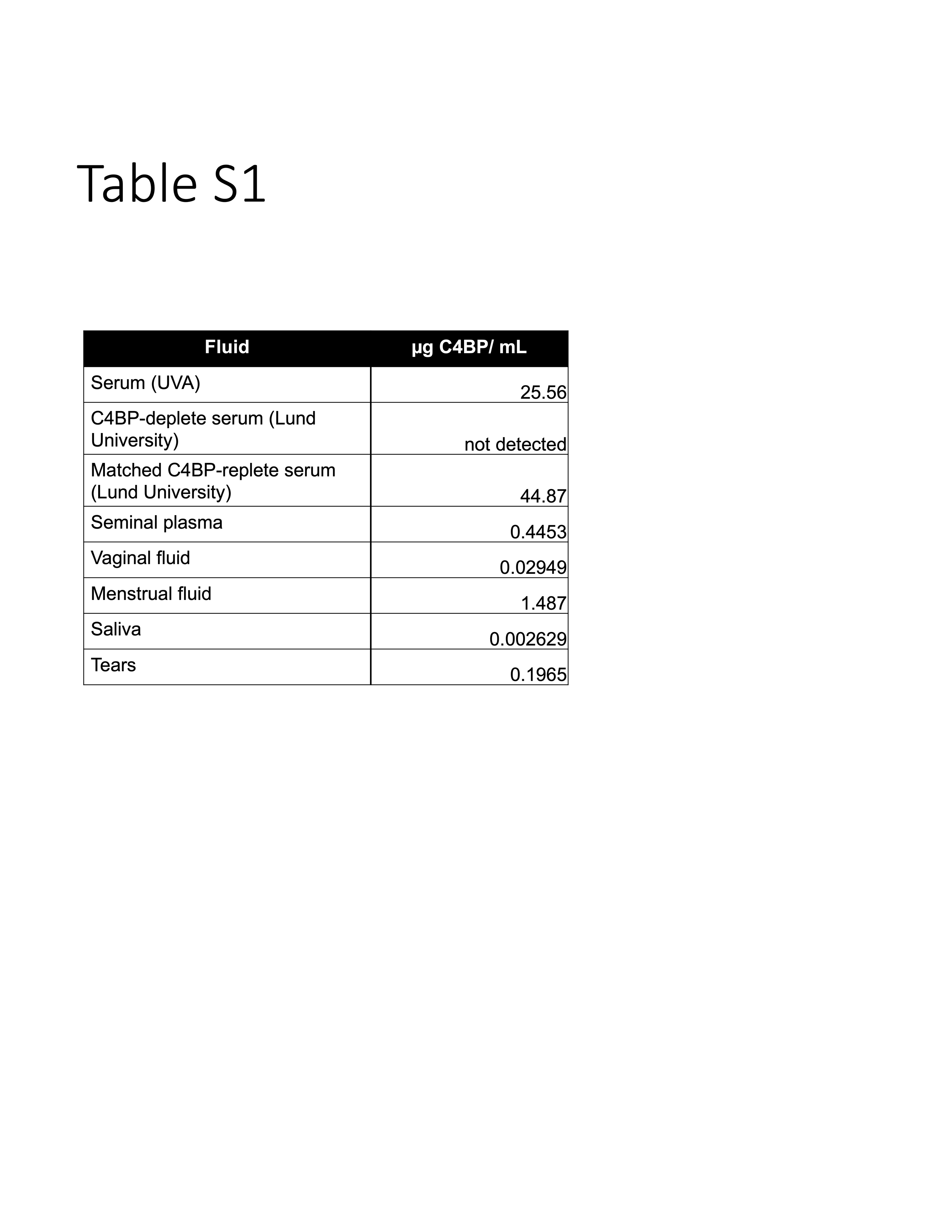

Supplement: S1 Table — (TIF) [file ppat.1011055.s010.tif]
